# Supplementary material for: Gradient and curl optical torques
Source: Nat Commun. 2024 Jul 24;15:6230. doi: 10.1038/s41467-024-50440-8 (PMC11266349; doi:10.1038/s41467-024-50440-8)
Supplement: Supplementary file 1 — Supplementary Information [file 41467_2024_50440_MOESM1_ESM.pdf]

Supplementary Information

Gradient and curl optical torques

Xiaohao Xu,[1,2] Manuel Nieto-Vesperinas,[3] Yuan Zhou,[1,2] Yanan Zhang,[1,2] Manman Li,[1,2] Francisco J. Rodríguez-Fortuño,[4,5] Shaohui Yan,[1,2] and Baoli Yao[1,2]

[1] *State Key Laboratory of Transient Optics and Photonics, Xi'an Institute of Optics and Precision Mechanics, Chinese Academy of Sciences, Xi'an 710119, China*

[2] *University of Chinese Academy of Sciences, Beijing 100049, China*

[3] *Instituto de Ciencia de Materiales de Madrid, Consejo Superior de Investigaciones Científicas, Campus de Cantoblanco, Madrid 28049, Spain.*

[4] *Department of Physics, King's College London, Strand, London WC2R 2LS, United Kingdom*

[5] *London Centre for Nanotechnology, Department of Physics, King's College London, Strand, London WC2R 2LS, United Kingdom*

Note 1. Multipole model of optical torque: an incident field-featured theory

It is known that both the optical force and torque can be calculated by the integral of Maxwell stress tensor (MST) of the total field [23]. While holding universally, the MST method, to some extent, conveys a black-box calculation procedure, because it implicates the scattered field which is usually unknown beforehand. That is why the dipole model is developed, as it describes the force and torque by incident fields and particle properties [7], thus making the problem more predictable and explainable. For higher multipoles, it was found that the torque can be expressed as a summation form involving the electric and magnetic multipole moments induced in the particle [42],

$$\mathbf{T} = \sum_{l=1}^{N} (\mathbf{T}_{\mathrm{mix}}^{(l)} + \mathbf{T}_{\mathrm{sca}}^{(l)}). \tag{1}$$

In Eq. (1), the extinction torque $\mathbf{T}_{\mathrm{mix}}^{(l)}$ represents the angular momentum removed per time from the incident fields by the electric (magnetic) 2^l-pole moment, while $\mathbf{T}_{\mathrm{sca}}^{(l)}$ is the recoiling torque arising from asymmetric scattering. They are given by

$$\begin{aligned} \mathbf{T}_{\mathrm{mix}}^{(l)} &= \tfrac{1}{2l!}\mathrm{Re}[(l-1)(\nabla^{(l-1)}\mathbf{E}^*) \overset{(l-1)}{\cdot\cdot} \mathbb{O}_{\mathrm{e}}^{(l)} - \mathbb{O}_{\mathrm{e}}^{(l)} \overset{(l-1)}{\cdot\cdot} (\nabla^{(l-1)}\mathbf{E}^*)] \overset{(2)}{\cdot\cdot} \boldsymbol{\epsilon}^{(3)} \\ &\quad + \tfrac{1}{2l!}\mathrm{Re}[(l-1)(\nabla^{(l-1)}\mathbf{B}^*) \overset{(l-1)}{\cdot\cdot} \mathbb{O}_{\mathrm{m}}^{(l)} - \mathbb{O}_{\mathrm{m}}^{(l)} \overset{(l-1)}{\cdot\cdot} (\nabla^{(l-1)}\mathbf{B}^*)] \overset{(2)}{\cdot\cdot} \boldsymbol{\epsilon}^{(3)}, \\ \mathbf{T}_{\mathrm{sca}}^{(l)} &= -\tfrac{k^{2l+1}2^l(l+1)}{8\pi\varepsilon(2l+1)!}\mathrm{Im}[\mathbb{O}_{\mathrm{e}}^{(l)} \overset{(l-1)}{\cdot\cdot} \mathbb{O}_{\mathrm{e}}^{(l)*} + \tfrac{1}{c^2}\mathbb{O}_{\mathrm{m}}^{(l)} \overset{(l-1)}{\cdot\cdot} \mathbb{O}_{\mathrm{m}}^{(l)*}] \overset{(2)}{\cdot\cdot} \boldsymbol{\epsilon}^{(3)}, \end{aligned} \tag{2}$$

with $\boldsymbol{\epsilon}^{(3)}$ referring to the Levi-Civita antisymmetric tensor of rank 3; for an isotropic sphere, the multipole moments are defined by

$$\mathbb{O}_{\mathrm{e(m)}}^{(l)} = \gamma_{\mathrm{e(m)}}^{(l)} \sum_{m=0}^{\lfloor (l-1)/2 \rfloor} d_{l,m} k^{2m} \hat{\mathcal{S}}[\mathbb{I}^{(m)} \otimes \mathbb{M}_{\mathrm{e(m)}}^{(l-2m)}], \tag{3}$$

with

$$\begin{gathered} d_{l,m} = \tfrac{(l-1)!}{4^m m!}\tfrac{\Gamma(l-m+1/2)}{\Gamma(l+1/2)\Gamma(l-2m)}, \\ \mathbb{I}^{(m)} = \overbrace{\mathbb{I} \otimes \mathbb{I} \cdots \otimes \mathbb{I}}^{m},\ \mathbb{M}_{\mathrm{e}}^{(k)} = \hat{\mathcal{S}}[\nabla^{(k-1)}\mathbf{E}],\ \mathbb{M}_{\mathrm{m}}^{(k)} = \hat{\mathcal{S}}[\nabla^{(k-1)}\mathbf{B}], \end{gathered} \tag{4}$$

and

$$\gamma_{\mathrm{e}}^{(l)} = \frac{4l(2l+1)!!}{(l+1)}\frac{i\pi\varepsilon a_l}{k^{2l+1}},\ \gamma_{\mathrm{m}}^{(l)} = \frac{4l(2l+1)!!}{(l+1)}\frac{i\pi b_l}{\mu k^{2l+1}}, \tag{5}$$

where a_l and b_l are Mie coefficients for an isotropic sphere. $\hat{\mathcal{S}}$ is the symmetrization operator, and $\lfloor x \rfloor$ represents the largest integer no larger than x. The cases of $l = 1, 2$ correspond to dipole and quadrupole moments,

$$\mathbb{O}_{\rm e}^{(1)} = \gamma_{\rm e}^{(1)}\mathbb{M}_{\rm e}^{(1)}, \mathbb{O}_{\rm e}^{(2)} = \gamma_{\rm e}^{(2)}\mathbb{M}_{\rm e}^{(2)}. \tag{6}$$

With the source-free condition, $\nabla \cdot \mathbf{E} = 0$, and the vector Helmholtz equation, $\nabla^2\mathbf{E} = -k^2\mathbf{E}$, we have

$$\begin{aligned}\mathbf{T}_{\rm mix}^{(l)} &= \frac{1}{2l!}\sum_{m=0}^{(l-1)/2} \mathrm{Re}\left\{\gamma_{\rm e}^{(l)}[\alpha_{l,m}\nabla^{(l-2m-1)}\mathbf{E}^* \overset{(l-2m-1)}{\cdot\cdot} \mathbb{M}_{\rm e}^{(l-2m)} - \chi_{l,m}\mathbb{M}_{\rm e}^{(l-2m)} \overset{(l-2m-1)}{\cdot\cdot} \nabla^{(l-2m-1)}\mathbf{E}^*]\right\} \overset{(2)}{\cdot\cdot} \boldsymbol{\epsilon}^{(3)} \\ &+ \frac{1}{2l!}\sum_{m=0}^{(l-1)/2} \mathrm{Re}\left\{\gamma_{\rm m}^{(l)}[\alpha_{l,m}\nabla^{(l-2m-1)}\mathbf{B}^* \overset{(l-2m-1)}{\cdot\cdot} \mathbb{M}_{\rm m}^{(l-2m)} - \chi_{l,m}\mathbb{M}_{\rm m}^{(l-2m)} \overset{(l-2m-1)}{\cdot\cdot} \nabla^{(l-2m-1)}\mathbf{B}^*]\right\} \overset{(2)}{\cdot\cdot} \boldsymbol{\epsilon}^{(3)},\end{aligned} \tag{7}$$

$$\mathbf{T}_{\rm sca}^{(l)} = -\frac{k^{2l+1}}{8\pi\varepsilon}\frac{2^l(l+1)}{(2l+1)!}\sum_{m=0}^{(l-1)/2}\beta_{l,m}\mathrm{Im}\left\{|\gamma_{\rm e}^{(l)}|[\mathbb{M}_{\rm e}^{(l-2m)} \overset{(l-2m-1)}{\cdot\cdot} \mathbb{M}_{\rm e}^{(l-2m)*}] + \frac{1}{c^2}|\gamma_{\rm m}^{(l)}|[\mathbb{M}_{\rm m}^{(l-2m)} \overset{(l-2m-1)}{\cdot\cdot} \mathbb{M}_{\rm m}^{(l-2m)*}]\right\} \overset{(2)}{\cdot\cdot} \boldsymbol{\epsilon}^{(3)}, \tag{8}$$

with

$$\alpha_{l,m} = (-1)^m k^{4m} d_{l,m}\frac{(l-2m)(l-2m-1)}{l}, \ \chi_{l,m} = (-1)^m k^{4m} d_{l,m}\frac{l-2m}{l}, \ \beta_{l,m}k^{4m}\frac{(-1)^m(l-2m)^2}{l^2}. \tag{9}$$

Eqs.(7) and (8) has expressed the torque in terms of incident field vectors. Our ultimate aim is to derive such a model from which the relation between the torque and field dynamic properties (e.g., spin) will be apparent, just like the well-known dipole model (see Eq.(1) in the main text). To proceed further, we turn to the momentum space, in which the incident fields at position $\mathbf{x}$ are represented as

$$\begin{aligned}\mathbf{E} = \int \mathbf{f}(\mathbf{n})e^{i\mathbf{k}\cdot\mathbf{x}}d\Omega, \quad \mathbf{B} = \int \mathbf{h}(\mathbf{n})e^{i\mathbf{k}\cdot\mathbf{x}}d\Omega \\ \mathbf{k}\times\mathbf{f}(\mathbf{n}) = \omega\mathbf{h}(\mathbf{n}), \quad \mathbf{k}\times\mathbf{h}(\mathbf{n}) = -(\omega/c^2)\mathbf{f}(\mathbf{n}), \quad \mathbf{n}\cdot\mathbf{f}(\mathbf{n}) = \mathbf{n}\cdot\mathbf{h}(\mathbf{n}) = 0,\end{aligned} \tag{10}$$

where Ω is a solid angle, and $\mathbf{n}$ is the unit vector denoting the direction of wave vector: $\mathbf{k} = k\mathbf{n}$. Eq. (10) is the angular spectrum representation of the wavefield. Plugging Eq.(10) into (7) and (8), the summand in each sigma takes a general form as

$$\iint [\mathbb{A}^{(n)*}(\mathbf{k}_1) \overset{(n-1)}{\cdot\cdot} \mathbb{B}^{(n)}(\mathbf{k}_2)] \overset{(2)}{\cdot\cdot} \boldsymbol{\epsilon}^{(3)}e^{-i\mathbf{k}_{12}\cdot\mathbf{x}}d\Omega, \tag{11}$$

with $\mathbf{k}_{12} = \mathbf{k}_1 - \mathbf{k}_2$. Specific to the summands in Eq.(7), we have

$$\begin{aligned}[(\nabla^{(l-2m-1)}\mathbf{E}^*) \overset{(l-2m-1)}{\cdot\cdot} \mathbb{M}_{\rm e}^{(l-2m)}] \overset{(2)}{\cdot\cdot} \boldsymbol{\epsilon}^{(3)} &\overset{\mathbf{k}}{=} [\mathbf{k}_1^{(l-2m-1)}\mathbf{f}_1^* \overset{(l-2m-1)}{\cdot\cdot} \hat{\mathcal{S}}(\mathbf{k}_2^{(l-2m-1)}\mathbf{f}_2)] \overset{(2)}{\cdot\cdot} \boldsymbol{\epsilon}^{(3)} \\ &= -\frac{(\mathbf{k}_1\cdot\mathbf{k}_2)^{(l-2m-3)}}{l-2m}\left\{\begin{array}{c}(\mathbf{k}_1\cdot\mathbf{k}_2)(\mathbf{k}_2\cdot\mathbf{f}_1^*)(\mathbf{k}_1\times\mathbf{f}_2)+ \\ (l-2m-2)(\mathbf{k}_2\cdot\mathbf{f}_1^*)(\mathbf{k}_1\cdot\mathbf{f}_2)(\mathbf{k}_1\times\mathbf{k}_2)+ \\ (\mathbf{k}_1\cdot\mathbf{k}_2)(\mathbf{f}_1^*\cdot\mathbf{f}_2)(\mathbf{k}_1\times\mathbf{k}_2)\end{array}\right\},\end{aligned} \tag{12}$$

(where $\overset{\mathbf{k}}{=}$ denotes taking the integrand of integral (Eq. (11)) without the exponent factor) and

$$\begin{aligned}[\mathbb{M}_{\rm e}^{(l-2m)} \overset{(l-2m-1)}{\cdot\cdot} (\nabla^{(l-2m-1)}\mathbf{E}^*)] \overset{(2)}{\cdot\cdot} \boldsymbol{\epsilon}^{(3)} &\overset{\mathbf{k}}{=} [\mathbf{k}_1^{(l-2m-1)}\mathbf{f}_1^* \overset{(l-2m-1)}{\cdot\cdot} \hat{\mathcal{S}}(\mathbf{k}_2^{(l-2m-1)}\mathbf{f}_2)] \overset{(2)}{\cdot\cdot} \boldsymbol{\epsilon}^{(3)} \\ &= -\frac{(\mathbf{k}_1\cdot\mathbf{k}_2)^{(l-2m-2)}}{l-2m}\left\{\begin{array}{c}(l-2m-1)(\mathbf{k}_1\cdot\mathbf{f}_2)(\mathbf{f}_1^*\times\mathbf{k}_2)+ \\ (\mathbf{k}_1\cdot\mathbf{k}_2)(\mathbf{f}_1^*\times\mathbf{f}_2)\end{array}\right\}.\end{aligned} \tag{13}$$

With replacement $\mathbf{f}\to\mathbf{h}$, similar expressions hold for summands in the magnetic part of Eq.(7):

$$\begin{aligned}[(\nabla^{(l-2m-1)}\mathbf{B}^*) \overset{(l-2m-1)}{\cdot\cdot} \mathbb{M}_{\rm m}^{(l-2m)}] \overset{(2)}{\cdot\cdot} \boldsymbol{\epsilon}^{(3)} &\overset{\mathbf{k}}{=} [\mathbf{k}_1^{(l-2m-1)}\mathbf{h}_1^* \overset{(l-2m-1)}{\cdot\cdot} \hat{\mathcal{S}}(\mathbf{k}_2^{(l-2m-1)}\mathbf{h}_2)] \overset{(2)}{\cdot\cdot} \boldsymbol{\epsilon}^{(3)} \\ &= -\frac{(\mathbf{k}_1\cdot\mathbf{k}_2)^{(l-2m-3)}}{l-2m}\left\{\begin{array}{c}(\mathbf{k}_1\cdot\mathbf{k}_2)(\mathbf{k}_2\cdot\mathbf{h}_1^*)(\mathbf{k}_1\times\mathbf{h}_2)+ \\ (l-2m-2)(\mathbf{k}_2\cdot\mathbf{h}_1^*)(\mathbf{k}_1\cdot\mathbf{h}_2)(\mathbf{k}_1\times\mathbf{k}_2)+ \\ (\mathbf{k}_1\cdot\mathbf{k}_2)(\mathbf{h}_1^*\cdot\mathbf{h}_2)(\mathbf{k}_1\times\mathbf{k}_2)\end{array}\right\},\end{aligned} \tag{14}$$

and

$$[\mathbb{M}_{\rm m}^{(l-2m)} \overset{(l-2m-1)}{\cdot\cdot} (\nabla^{(l-2m-1)}\mathbf{B}^*)] \overset{(2)}{\cdot\cdot} \boldsymbol{\epsilon}^{(3)} \overset{\mathbf{k}}{=} [\mathbf{k}_1^{(l-2m-1)}\mathbf{h}_1^* \overset{(l-2m-1)}{\cdot\cdot} \hat{\mathcal{S}}(\mathbf{k}_2^{(l-2m-1)}\mathbf{h}_2)] \overset{(2)}{\cdot\cdot} \boldsymbol{\epsilon}^{(3)}$$
$$= -\frac{(\mathbf{k}_1\cdot\mathbf{k}_2)^{(l-2m-2)}}{l-2m}\left\{\begin{array}{c} (l-2m-1)(\mathbf{k}_1\cdot\mathbf{h}_2)(\mathbf{h}_1^*\times\mathbf{k}_2)+ \\ (\mathbf{k}_1\cdot\mathbf{k}_2)(\mathbf{h}_1^*\times\mathbf{h}_2) \end{array}\right\}. \tag{15}$$

For the summands in Eq.(8), we have

$$[\mathbb{M}_{\rm e}^{(l-2m)} \overset{(l-2m-1)}{\cdot\cdot} \mathbb{M}_{\rm e}^{(l-2m)*}] \overset{(2)}{\cdot\cdot} \boldsymbol{\epsilon}^{(3)} \overset{\mathbf{k}}{=} [\hat{\mathcal{S}}(\mathbf{k}_2^{(l-2m-1)}\mathbf{f}_2) \overset{(l-2m-1)}{\cdot\cdot} \hat{\mathcal{S}}(\mathbf{k}_1^{(l-2m-1)}\mathbf{f}_1^*)] \overset{(2)}{\cdot\cdot} \boldsymbol{\epsilon}^{(3)}$$
$$= -\frac{(\mathbf{k}_1\cdot\mathbf{k}_2)^{(l-2m-3)}}{(l-2m)^2}\left\{\begin{array}{c} (\mathbf{k}_1\cdot\mathbf{k}_2)^2(\mathbf{f}_1^*\times\mathbf{f}_2)+ \\ (l-2m-1)(\mathbf{k}_1\cdot\mathbf{k}_2)(\mathbf{k}_1\cdot\mathbf{f}_2)(\mathbf{f}_1^*\times\mathbf{k}_2)+ \\ (l-2m-1)(\mathbf{k}_1\cdot\mathbf{k}_2)(\mathbf{k}_2\cdot\mathbf{f}_1^*)(\mathbf{k}_1\times\mathbf{f}_2) \\ (l-2m-1)(\mathbf{k}_1\cdot\mathbf{k}_2)(\mathbf{f}_1^*\cdot\mathbf{f}_2)(\mathbf{k}_1\times\mathbf{k}_2) \\ (l-2m-1)(l-2m-2)(\mathbf{k}_2\cdot\mathbf{f}_1^*)(\mathbf{k}_1\cdot\mathbf{f}_2)(\mathbf{k}_1\times\mathbf{k}_2) \end{array}\right\}, \tag{16}$$

and

$$[\mathbb{M}_{\rm m}^{(l-2m)} \overset{(l-2m-1)}{\cdot\cdot} \mathbb{M}_{\rm m}^{(l-2m)*}] \overset{(2)}{\cdot\cdot} \boldsymbol{\epsilon}^{(3)} \overset{\mathbf{k}}{=} [\hat{\mathcal{S}}(\mathbf{k}_2^{(l-2m-1)}\mathbf{h}_2) \overset{(l-2m-1)}{\cdot\cdot} \hat{\mathcal{S}}(\mathbf{k}_1^{(l-2m-1)}\mathbf{h}_1^*)] \overset{(2)}{\cdot\cdot} \boldsymbol{\epsilon}^{(3)}$$
$$= -\frac{(\mathbf{k}_1\cdot\mathbf{k}_2)^{(l-2m-3)}}{(l-2m)^2}\left\{\begin{array}{c} (\mathbf{k}_1\cdot\mathbf{k}_2)^2(\mathbf{h}_1^*\times\mathbf{h}_2)+ \\ (l-2m-1)(\mathbf{k}_1\cdot\mathbf{k}_2)(\mathbf{k}_1\cdot\mathbf{h}_2)(\mathbf{h}_1^*\times\mathbf{k}_2)+ \\ (l-2m-1)(\mathbf{k}_1\cdot\mathbf{k}_2)(\mathbf{k}_2\cdot\mathbf{h}_1^*)(\mathbf{k}_1\times\mathbf{h}_2) \\ (l-2m-1)(\mathbf{k}_1\cdot\mathbf{k}_2)(\mathbf{h}_1^*\cdot\mathbf{h}_2)(\mathbf{k}_1\times\mathbf{k}_2) \\ (l-2m-1)(l-2m-2)(\mathbf{k}_2\cdot\mathbf{h}_1^*)(\mathbf{k}_1\cdot\mathbf{h}_2)(\mathbf{k}_1\times\mathbf{k}_2) \end{array}\right\}. \tag{17}$$

We list some useful relations for later calculations:

$$\begin{aligned}(\mathbf{f}_1^*\cdot\mathbf{k}_2)(\mathbf{k}_1\times\mathbf{f}_2) &= [(\mathbf{f}_1^*\cdot\mathbf{k}_2)\mathbf{k}_1]\times\mathbf{f}_2 \\ &= [\mathbf{k}_2\times(\mathbf{k}_1\times\mathbf{f}_1^*)+\mathbf{f}_1^*(\mathbf{k}_1\cdot\mathbf{k}_2)]\times\mathbf{f}_2 \\ &= \omega(\mathbf{k}_2\times\mathbf{h}_1^*)\times\mathbf{f}_2+(\mathbf{k}_1\cdot\mathbf{k}_2)(\mathbf{f}_1^*\times\mathbf{f}_2) \\ &= -\omega\mathbf{k}_2(\mathbf{h}_1^*\cdot\mathbf{f}_2)+(\mathbf{k}_1\cdot\mathbf{k}_2)(\mathbf{f}_1^*\times\mathbf{f}_2) \\ &= (\mathbf{k}_1\cdot\mathbf{k}_2)(\mathbf{f}_1^*\times\mathbf{f}_2)+\frac{1}{2}[\omega\mathbf{k}_{12}(\mathbf{h}_1^*\cdot\mathbf{f}_2)-k^2\mathbf{f}_1^*\times\mathbf{f}_2-\omega^2\mathbf{h}_1^*\times\mathbf{h}_2-\omega\mathbf{k}_{12}\times(\mathbf{h}_1^*\times\mathbf{f}_2)],\end{aligned} \tag{18}$$

$$\begin{aligned}(\mathbf{k}_2\cdot\mathbf{f}_1^*)(\mathbf{k}_1\cdot\mathbf{f}_2)(\mathbf{k}_1\times\mathbf{k}_2) &= [(\mathbf{k}_1\cdot\mathbf{k}_2)(\mathbf{f}_1^*\cdot\mathbf{f}_2)-(\mathbf{k}_1\times\mathbf{f}_1^*)\cdot(\mathbf{k}_2\times\mathbf{f}_2)](\mathbf{k}_1\times\mathbf{k}_2) \\ &= [(\mathbf{k}_1\cdot\mathbf{k}_2)(\mathbf{f}_1^*\cdot\mathbf{f}_2)-\omega^2(\mathbf{h}_1^*\cdot\mathbf{h}_2)](\mathbf{k}_1\times\mathbf{k}_2) \\ &= \left\{\begin{array}{c} [(\mathbf{k}_1\cdot\mathbf{k}_2)-k^2][(\mathbf{k}_1\cdot\mathbf{k}_2)(\mathbf{f}_1^*\times\mathbf{f}_2)-\omega^2(\mathbf{h}_1^*\times\mathbf{h}_2)]+ \\ \frac{\omega}{2}\mathbf{k}_{12}[(\mathbf{k}_1\cdot\mathbf{k}_2)+k^2][(\mathbf{f}_1^*\cdot\mathbf{h}_2)+(\mathbf{h}_1^*\cdot\mathbf{f}_2)]+ \\ \frac{\omega}{2}[(\mathbf{k}_1\cdot\mathbf{k}_2)-k^2]\mathbf{k}_{12}\times[(\mathbf{f}_1^*\times\mathbf{h}_2)-(\mathbf{h}_1^*\times\mathbf{f}_2)] \end{array}\right\},\end{aligned} \tag{19}$$

$$\begin{aligned}(\mathbf{f}_1^*\cdot\mathbf{f}_2)(\mathbf{k}_1\times\mathbf{k}_2) &= [(\mathbf{f}_1^*\cdot\mathbf{f}_2)\mathbf{k}_1]\times\mathbf{k}_2 = [\mathbf{f}_2\times(\mathbf{k}_1\times\mathbf{f}_1^*)+\mathbf{f}_1^*(\mathbf{k}_1\cdot\mathbf{f}_2)]\times\mathbf{k}_2 \\ &= \omega(\mathbf{f}_2\times\mathbf{h}_1^*)\times\mathbf{k}_2+(\mathbf{k}_1\cdot\mathbf{f}_2)(\mathbf{f}_1^*\times\mathbf{k}_2) \\ &= -\omega\mathbf{f}_2(\mathbf{k}_2\cdot\mathbf{h}_1^*)+\mathbf{f}_1^*\times[(\mathbf{k}_1\cdot\mathbf{f}_2)\mathbf{k}_2] \\ &= -\omega\mathbf{f}_2(\mathbf{k}_2\cdot\mathbf{h}_1^*)+\mathbf{f}_1^*\times[\mathbf{k}_1\times(\mathbf{k}_2\times\mathbf{f}_2)+\mathbf{f}_2(\mathbf{k}_1\cdot\mathbf{k}_2)] \\ &= \omega[\mathbf{k}_1(\mathbf{f}_1^*\cdot\mathbf{h}_2)-\mathbf{f}_2(\mathbf{k}_2\cdot\mathbf{h}_1^*)]+(\mathbf{k}_1\cdot\mathbf{k}_2)(\mathbf{f}_1^*\times\mathbf{f}_2) \\ &= (\mathbf{k}_1\cdot\mathbf{k}_2)(\mathbf{f}_1^*\times\mathbf{f}_2)+\omega^2(\mathbf{h}_1^*\cdot\mathbf{h}_2)+\omega\mathbf{k}_1(\mathbf{f}_1^*\cdot\mathbf{h}_2)-\omega\mathbf{k}_2(\mathbf{h}_1^*\cdot\mathbf{f}_2) \\ &= [(\mathbf{k}_1\cdot\mathbf{k}_2)-k^2](\mathbf{f}_1^*\times\mathbf{f}_2)+\frac{\omega}{2}\mathbf{k}_{12}[(\mathbf{f}_1^*\cdot\mathbf{h}_2)+(\mathbf{h}_1^*\cdot\mathbf{f}_2)]+\frac{\omega}{2}\mathbf{k}_{12}\times[(\mathbf{f}_1^*\times\mathbf{h}_2)-(\mathbf{h}_1^*\times\mathbf{f}_2)].\end{aligned} \tag{20}$$

To simplify writing in subsequent calculations, we introduce the following notations:

$$\begin{gathered}\boldsymbol{\mathcal{S}}_{\rm ee}\equiv\mathbf{f}_1^*\times\mathbf{f}_2,\quad \boldsymbol{\mathcal{S}}_{\rm mm}\equiv\mathbf{h}_1^*\times\mathbf{h}_2,\quad \boldsymbol{\mathcal{S}}_{\rm em}\equiv\mathbf{f}_1^*\times\mathbf{h}_2, \\ \boldsymbol{\mathcal{S}}_{\rm me}\equiv\mathbf{h}_1^*\times\mathbf{f}_2,\quad \mathcal{D}_{\rm em}\equiv\mathbf{f}_1^*\cdot\mathbf{h}_2,\quad \mathcal{D}_{\rm me}\equiv\mathbf{h}_1^*\cdot\mathbf{f}_2.\end{gathered} \tag{21}$$

Upon substitution from Eqs. (18)-(21) to Eqs. (12)-(17), we obtain

$$
\begin{aligned}
&[\alpha_{l,m}\nabla^{(l-2m-1)}\mathbf{E}^{*} \overset{(l-2m-1)}{\cdot\cdot} \mathbb{M}_{\mathrm{e}}^{(l-2m)} - \chi_{l,m}\mathbb{M}_{\mathrm{e}}^{(l-2m)} \overset{(l-2m-1)}{\cdot\cdot} \nabla^{(l-2m-1)}\mathbf{E}^{*}] \overset{(2)}{\cdot\cdot} \boldsymbol{\epsilon}^{(3)} \\
&\overset{\mathbf{k}}{=} \chi_{l,m}\frac{(\mathbf{k}_1\cdot\mathbf{k}_2)^{(l-2m-3)}}{2(l-2m)}\left\{\begin{array}{c}
(2l-4m)[k^2(l-2m-1)(\mathbf{k}_1\cdot\mathbf{k}_2)-(l-2m)(\mathbf{k}_1\cdot\mathbf{k}_2)^2]\boldsymbol{\mathcal{S}}_{\mathrm{ee}} \\
+2\omega^2(l-2m-1)[(l-2m-1)(\mathbf{k}_1\cdot\mathbf{k}_2)-k^2(l-2m-2)]\boldsymbol{\mathcal{S}}_{\mathrm{mm}} \\
+\omega(l-2m-1)[k^2(l-2m-2)-(l-2m)(\mathbf{k}_1\cdot\mathbf{k}_2)]\mathbf{k}_{12}\times(\boldsymbol{\mathcal{S}}_{\mathrm{em}}-\boldsymbol{\mathcal{S}}_{\mathrm{me}}) \\
-\omega(l-2m-1)[k^2(l-2m-2)+(l-2m)(\mathbf{k}_1\cdot\mathbf{k}_2)]\mathbf{k}_{12}(\mathcal{D}_{\mathrm{em}}+\mathcal{D}_{\mathrm{me}})
\end{array}\right\},
\end{aligned}
\tag{22}
$$

$$
\begin{aligned}
&[\alpha_{l,m}\nabla^{(l-2m-1)}\mathbf{B}^{*} \overset{(l-2m-1)}{\cdot\cdot} \mathbb{M}_{\mathrm{m}}^{(l-2m)} - \chi_{l,m}\mathbb{M}_{\mathrm{m}}^{(l-2m)} \overset{(l-2m-1)}{\cdot\cdot} \nabla^{(l-2m-1)}\mathbf{B}^{*}] \overset{(2)}{\cdot\cdot} \boldsymbol{\epsilon}^{(3)} \\
&\overset{\mathbf{k}}{=} \chi_{l,m}\frac{(\mathbf{k}_1\cdot\mathbf{k}_2)^{(l-2m-3)}}{2(l-2m)\omega^2}\left\{\begin{array}{c}
\omega^2(2l-4m)[k^2(l-2m-1)(\mathbf{k}_1\cdot\mathbf{k}_2)-(l-2m)(\mathbf{k}_1\cdot\mathbf{k}_2)^2]\boldsymbol{\mathcal{S}}_{\mathrm{mm}} \\
+2k^4(l-2m-1)[(l-2m-1)(\mathbf{k}_1\cdot\mathbf{k}_2)-k^2(l-2m-2)]\boldsymbol{\mathcal{S}}_{\mathrm{ee}} \\
+\omega k^2(l-2m-1)[k^2(l-2m-2)-(l-2m)(\mathbf{k}_1\cdot\mathbf{k}_2)]\mathbf{k}_{12}\times(\boldsymbol{\mathcal{S}}_{\mathrm{em}}-\boldsymbol{\mathcal{S}}_{\mathrm{me}}) \\
+\omega k^2(l-2m-1)[k^2(l-2m-2)+(l-2m)(\mathbf{k}_1\cdot\mathbf{k}_2)]\mathbf{k}_{12}(\mathcal{D}_{\mathrm{em}}+\mathcal{D}_{\mathrm{me}})
\end{array}\right\},
\end{aligned}
\tag{23}
$$

$$
\begin{aligned}
&[\mathbb{M}_{\mathrm{e}}^{(l-2m)} \overset{(l-2m-1)}{\cdot\cdot} \mathbb{M}_{\mathrm{e}}^{(l-2m)*}] \overset{(2)}{\cdot\cdot} \boldsymbol{\epsilon}^{(3)} \\
&\overset{\mathbf{k}}{=} \frac{(\mathbf{k}_1\cdot\mathbf{k}_2)^{l-2m-3}}{2(l-2m)^2}\left\{\begin{array}{c}
2[(l-2m)^2(\mathbf{k}_1\cdot\mathbf{k}_2)^2-k^2(l-2m)(l-2m-1)(\mathbf{k}_1\cdot\mathbf{k}_2)]\boldsymbol{\mathcal{S}}_{\mathrm{ee}} \\
+2\omega^2(l-2m-1)[k^2(l-2m-2)-(l-2m-1)(\mathbf{k}_1\cdot\mathbf{k}_2)]\boldsymbol{\mathcal{S}}_{\mathrm{mm}} \\
+\omega(l-2m-1)[(l-2m)(\mathbf{k}_1\cdot\mathbf{k}_2)-k^2(l-2m-2)]\mathbf{k}_{12}\times(\boldsymbol{\mathcal{S}}_{\mathrm{em}}-\boldsymbol{\mathcal{S}}_{\mathrm{me}}) \\
+\omega(l-2m-1)[(l-2m)(\mathbf{k}_1\cdot\mathbf{k}_2)+k^2(l-2m-2)]\mathbf{k}_{12}(\mathcal{D}_{\mathrm{em}}+\mathcal{D}_{\mathrm{me}})
\end{array}\right\},
\end{aligned}
\tag{24}
$$

$$
\begin{aligned}
&[\mathbb{M}_{\mathrm{mag}}^{(l-2m)} \overset{(l-2m-1)}{\cdot\cdot} \mathbb{M}_{\mathrm{mag}}^{(l-2m)*}] \overset{(2)}{\cdot\cdot} \boldsymbol{\epsilon}^{(3)} \\
&\overset{\mathbf{k}}{=} \frac{(\mathbf{k}_1\cdot\mathbf{k}_2)^{l-2m-3}}{2(l-2m)^2\omega^2}\left\{\begin{array}{c}
2\omega^2[(l-2m)^2(\mathbf{k}_1\cdot\mathbf{k}_2)^2-k^2(l-2m)(l-2m-1)(\mathbf{k}_1\cdot\mathbf{k}_2)]\boldsymbol{\mathcal{S}}_{\mathrm{mm}} \\
+2k^4(l-2m-1)[k^2(l-2m-2)-(l-2m-1)(\mathbf{k}_1\cdot\mathbf{k}_2)]\boldsymbol{\mathcal{S}}_{\mathrm{ee}} \\
+\omega k^2(l-2m-1)[(l-2m)(\mathbf{k}_1\cdot\mathbf{k}_2)-k^2(l-2m-2)]\mathbf{k}_{12}\times(\boldsymbol{\mathcal{S}}_{\mathrm{em}}-\boldsymbol{\mathcal{S}}_{\mathrm{me}}) \\
-\omega k^2(l-2m-1)[(l-2m)(\mathbf{k}_1\cdot\mathbf{k}_2)+k^2(l-2m-2)]\mathbf{k}_{12}(\mathcal{D}_{\mathrm{em}}+\mathcal{D}_{\mathrm{me}})
\end{array}\right\}.
\end{aligned}
\tag{25}
$$

Performing a Fourier transform to real-space for Eqs. (22)-(25) yields:

$$
\begin{aligned}
&[\alpha_{l,m}\nabla^{(l-2m-1)}\mathbf{E}^{*} \overset{(l-2m-1)}{\cdot\cdot} \mathbb{M}_{\mathrm{e}}^{(l-2m)} - \chi_{l,m}\mathbb{M}_{\mathrm{e}}^{(l-2m)} \overset{(l-2m-1)}{\cdot\cdot} \nabla^{(l-2m-1)}\mathbf{E}^{*}] \overset{(2)}{\cdot\cdot} \boldsymbol{\epsilon}^{(3)} \\
&= \frac{(-1)^m k^{4m} d_{l,m}}{l}\left\{\begin{array}{c}
(l-2m)[k^2(l-2m-1)\mathbf{S}_{\mathrm{ee}}^{(n-1)}-(l-2m)\mathbf{S}_{\mathrm{ee}}^{(n)}] \\
+\omega^2(l-2m-1)[(l-2m-1)\mathbf{S}_{\mathrm{mm}}^{(l-2m-1)}-k^2(l-2m-2)\mathbf{S}_{\mathrm{mm}}^{(l-2m-2)}] \\
+i\omega(l-2m-1)[k^2(l-2m-2)\nabla\times\mathrm{Re}\mathbf{S}_{\mathrm{em}}^{(l-2m-2)}-(l-2m)\nabla\times\mathrm{Re}\mathbf{S}_{\mathrm{em}}^{(l-2m-1)}] \\
-i\omega(l-2m-1)[k^2(l-2m-2)\nabla\mathrm{Re}D_{\mathrm{em}}^{(l-2m-2)}+(l-2m)\nabla\mathrm{Re}D_{\mathrm{em}}^{(l-2m-1)}]
\end{array}\right\},
\end{aligned}
\tag{26}
$$

$$
\begin{aligned}
&[\alpha_{l,m}\nabla^{(l-2m-1)}\mathbf{B}^{*} \overset{(l-2m-1)}{\cdot\cdot} \mathbb{M}_{\mathrm{m}}^{(l-2m)} - \chi_{l,m}\mathbb{M}_{\mathrm{m}}^{(l-2m)} \overset{(l-2m-1)}{\cdot\cdot} \nabla^{(l-2m-1)}\mathbf{B}^{*}] \overset{(2)}{\cdot\cdot} \boldsymbol{\epsilon}^{(3)} \\
&= \frac{(-1)^m k^{4m} d_{l,m}}{\omega^2 l}\left\{\begin{array}{c}
\omega^2(l-2m)[k^2(l-2m-1)\mathbf{S}_{\mathrm{mm}}^{(l-2m-1)}-(l-2m)\mathbf{S}_{\mathrm{mm}}^{(l-2m)}] \\
+k^4(l-2m-1)[(l-2m-1)\mathbf{S}_{\mathrm{ee}}^{(l-2m-1)}-k^2(l-2m-2)\mathbf{S}_{\mathrm{ee}}^{(l-2m-2)}] \\
+i\omega k^2(l-2m-1)[k^2(l-2m-2)\nabla\times\mathrm{Re}\mathbf{S}_{\mathrm{em}}^{(l-2m-2)}-(l-2m)\nabla\times\mathrm{Re}\mathbf{S}_{\mathrm{em}}^{(l-2m-1)}] \\
+i\omega k^2(l-2m-1)[k^2(l-2m-2)\nabla\mathrm{Re}D_{\mathrm{em}}^{(l-2m-2)}+(l-2m)\nabla\mathrm{Re}D_{\mathrm{em}}^{(l-2m-1)}]
\end{array}\right\},
\end{aligned}
\tag{27}
$$

$$
\begin{aligned}
&[\mathbb{M}_{\mathrm{e}}^{(l-2m)} \overset{(l-2m-1)}{\cdot\cdot} \mathbb{M}_{\mathrm{e}}^{(l-2m)*}] \overset{(2)}{\cdot\cdot} \boldsymbol{\epsilon}^{(3)} \\
&= \frac{1}{(l-2m)^2}\left\{\begin{array}{c}
[(l-2m)^2\mathbf{S}_{\mathrm{ee}}^{(l-2m)}-k^2(l-2m)(l-2m-1)\mathbf{S}_{\mathrm{ee}}^{(l-2m-1)}] \\
+\omega^2(l-2m-1)[k^2(l-2m-2)\mathbf{S}_{\mathrm{mm}}^{(l-2m-2)}-(l-2m-1)\mathbf{S}_{\mathrm{mm}}^{(l-2m-1)}] \\
+i\omega(l-2m-1)[(l-2m)\nabla\times\mathrm{Re}\mathbf{S}_{\mathrm{em}}^{(l-2m-1)}-k^2(l-2m-2)\nabla\times\mathrm{Re}\mathbf{S}_{\mathrm{em}}^{(l-2m-2)}] \\
+i\omega(l-2m-1)[(l-2m)\nabla\mathrm{Re}D_{\mathrm{em}}^{(l-2m-1)}+k^2(l-2m-2)\nabla\mathrm{Re}D_{\mathrm{em}}^{(l-2m-2)}]
\end{array}\right\},
\end{aligned}
\tag{28}
$$

$$[\mathbb{M}_{\text{mag}}^{(l-2m)} \overset{(l-2m-1)}{\cdot\cdot} \mathbb{M}_{\text{mag}}^{(l-2m)*}] \overset{(2)}{\cdot\cdot} \boldsymbol{\epsilon}^{(3)}$$
$$= \frac{1}{\omega^2(l-2m)^2} \left\{ \begin{array}{c} \omega^2[(l-2m)^2\mathbf{S}_{\text{mm}}^{(l-2m)} - k^2(l-2m)(l-2m-1)\mathbf{S}_{\text{mm}}^{(l-2m-1)}] \\ +k^4(l-2m-1)[k^2(l-2m-2)\mathbf{S}_{\text{ee}}^{(l-2m-2)} - (l-2m-1)\mathbf{S}_{\text{ee}}^{(l-2m-1)}] \\ +i\omega k^2(l-2m-1)[(l-2m)\nabla\times\text{Re}\mathbf{S}_{\text{em}}^{(l-2m-1)} - k^2(l-2m-2)\nabla\times\text{Re}\mathbf{S}_{\text{em}}^{(l-2m-2)}] \\ -i\omega k^2(l-2m-1)[(l-2m)\nabla\text{Re}D_{\text{em}}^{(l-2m-1)} + k^2(l-2m-2)\nabla\text{Re}D_{\text{em}}^{(l-2m-2)}] \end{array} \right\}, \tag{29}$$

where we have constructed the field moments,

$$\begin{aligned} \mathbf{S}_{\text{ee}}^{(n)} &= [(\nabla^{(n-1)}\mathbf{E}^*) \overset{(n-1)}{:} (\nabla^{(n-1)}\mathbf{E})] \overset{(2)}{:} \boldsymbol{\epsilon}^{(3)}, \\ \mathbf{S}_{\text{mm}}^{(n)} &= [(\nabla^{(n-1)}\mathbf{B}^*) \overset{(n-1)}{:} (\nabla^{(n-1)}\mathbf{B})] \overset{(2)}{:} \boldsymbol{\epsilon}^{(3)}, \\ \mathbf{S}_{\text{em}}^{(n)} &= [(\nabla^{(n-1)}\mathbf{E}^*) \overset{(n-1)}{:} (\nabla^{(n-1)}\mathbf{B})] \overset{(2)}{:} \boldsymbol{\epsilon}^{(3)}, \\ D_{\text{em}}^{(n)} &= [(\nabla^{(n-1)}\mathbf{E}^*) \overset{(n)}{:} (\nabla^{(n-1)}\mathbf{B})]. \end{aligned} \tag{30}$$

Employing the order reduction method introduced in [8], one may recast these quantities into physically apparent forms:

$$\begin{aligned} \mathbf{S}_{\text{ee}}^{(n)} &= (k^2 + \frac{\Delta}{2})^{n-1}(\mathbf{E}^* \times \mathbf{E}), \\ \mathbf{S}_{\text{mm}}^{(n)} &= (k^2 + \frac{\Delta}{2})^{n-1}(\mathbf{B}^* \times \mathbf{B}), \\ \mathbf{S}_{\text{em}}^{(n)} &= (k^2 + \frac{\Delta}{2})^{n-1}(\mathbf{E}^* \times \mathbf{B}), \\ D_{\text{em}}^{(n)} &= (k^2 + \frac{\Delta}{2})^{n-1}(\mathbf{E}^* \cdot \mathbf{B}). \end{aligned} \tag{31}$$

It is evident that these field moments originate from the optical spin, complex Poynting momentum and complex helicity. Finally, upon substitution of Eqs.(26)-(29),(31) into Eqs. (7) and (8), one may decompose the 2^l-pole torque into three parts according to field-related quantities:

$$\mathbf{T}^{(l)} = \mathbf{T}_{\text{spin}}^{(l)} + \mathbf{T}_{\text{grad}}^{(l)} + \mathbf{T}_{\text{curl}}^{(l)} = [\hat{\mathcal{A}}_{\text{e}}^{(l)}\mathbf{s}_{\text{e}} + \hat{\mathcal{A}}_{\text{m}}^{(l)}\mathbf{s}_{\text{m}}] + \hat{\mathcal{A}}_{\text{grad}}^{(l)}\nabla\mathcal{H} + \hat{\mathcal{A}}_{\text{curl}}^{(l)}\nabla\times\mathbf{p}, \tag{32}$$

where

$$\begin{aligned} \hat{\mathcal{A}}_{\text{e}}^{(l)} &= \sum_{m=0}^{\lfloor (l-1)/2 \rfloor} f_{l,m}\{C_{\text{a-e}}^{(l)}(l-2m)[\hat{\Delta}^{(l-2m)} - \hat{\Delta}^{(l-2m-1)}] + C_{\text{a-m}}^{(l)}(l-2m-1)[\hat{\Delta}^{(l-2m-1)} - \hat{\Delta}^{(l-2m-2)}]\}, \\ \hat{\mathcal{A}}_{\text{m}}^{(l)} &= \sum_{m=0}^{\lfloor (l-1)/2 \rfloor} f_{l,m}\{C_{\text{a-m}}^{(l)}(l-2m)[\hat{\Delta}^{(l-2m)} - \hat{\Delta}^{(l-2m-1)}] + C_{\text{a-e}}^{(l)}(l-2m-1)[\hat{\Delta}^{(l-2m-1)} - \hat{\Delta}^{(l-2m-2)}]\}, \\ \hat{\mathcal{A}}_{\text{grad}}^{(l)} &= \sum_{m=0}^{\lfloor (l-1)/2 \rfloor} \frac{f_{l,m}}{k}[C_{\text{a-e}}^{(l)} - C_{\text{a-m}}^{(l)}][(l-2m)\hat{\Delta}^{(l-2m-1)} + (l-2m-1)\hat{\Delta}^{(l-2m-2)}], \\ \hat{\mathcal{A}}_{\text{curl}}^{(l)} &= \sum_{m=0}^{\lfloor (l-1)/2 \rfloor} \frac{f_{l,m}}{k^2}[C_{\text{a-e}}^{(l)} + C_{\text{a-m}}^{(l)}][(l-2m)\hat{\Delta}^{(l-2m-1)} - (l-2m-1)\hat{\Delta}^{(l-2m-2)}]. \end{aligned} \tag{33}$$

We have introduced the parameter and operator:

$$f_{l,m} = c\frac{(2l-1)!!}{l(l+1)}\frac{(-1)^m}{4^m m!}\frac{\Gamma(l-m+1/2)}{\Gamma(l+1/2)\Gamma(l-2m)}, \quad \hat{\Delta}^{(n)} = n(1+\frac{\Delta}{2k^2})^{n-1}. \tag{34}$$

The absorption cross-sections due to electric and magnetic 2^l-poles are defined by:

$$C_{\text{a-e}}^{(l)} = \frac{2\pi}{k^2}(2l+1)[\text{Re}(a_l) - |a_l|^2], \quad C_{\text{a-m}}^{(l)} = \frac{2\pi}{k^2}(2l+1)[\text{Re}(b_l) - |b_l|^2]. \tag{35}$$

For $l=1$, there is only a single summand in Eq.(33), which corresponds to $m=0$. Then we arrive at $l-2m-1=0$ and $\hat{\Delta}^{(l-2m-1)}=\hat{\Delta}^{(0)}=0$, which indicates $\hat{\mathcal{A}}^{(l=1)}_{\text{grad}}=\hat{\mathcal{A}}^{(l=1)}_{\text{curl}}=0$, i.e., the gradient and curl torques being zero for the dipoles.

Note that each component in Eq. (32) can be produced by both the processes of light extinction and scattering (or recoiling effects). One may obtain their extinction and recoiling parts by decomposing the absorption cross-sections in Eq. (33) into the extinction and scattering cross-sections. In addition, as is clear from Eqs. (33) and (2), the torque generation does not concern the interplay between different multipoles, in contrast to the force for which the multipole interplay yields its recoiling part [7,8,42,43].

Note 2. Optical torque on nonspherical particles

In this note we present a rigorous argument proving that the nonconservative component T_z^{ncons} in Fig. 3 (or the travelling wave) is traced to the momentum curl. We will also show analytically that this T_z^{ncons} is attributed to the gradient of reactive helicity if the wave becomes evanescent. For a dipolar dimer or other rotationally symmetrical structures (e.g., cylinders and spheroids), the torque can be derived by replacing the scalar polarizability, i.e., Eq. (3), by a tensor, as previous work demonstrated [52]. However, such a treatment is invalid for anisotropic higher multipoles, because it will break the symmetric and traceless properties of multipole moments, which are required by definition [42]. To address the anisotropy, we employ a generic expression of the torque [55]:

$$T_z=-\frac{\varepsilon}{2k^3}\sum_{l=1}^{N}\sum_{m=-l}^{l}m[\text{Re}(q_{ml}b^*_{ml}+p_{ml}a^*_{ml})+|q_{ml}|^2+|p_{ml}|^2] \tag{36}$$

where p_{ml} and q_{ml} are the expansion coefficients of the scattered field on the basis of vector spherical wave functions (VSWFs). They are linked to the expansion coefficients of the incident field, a_{ml} and b_{ml}, through the so-called T matrix [54]:

$$\begin{bmatrix}\mathsf{a}\\ \mathsf{b}\end{bmatrix}=\mathsf{T}\begin{bmatrix}\mathsf{p}\\ \mathsf{q}\end{bmatrix}=\begin{bmatrix}\mathsf{T}^{11} & \mathsf{T}^{12}\\ \mathsf{T}^{21} & \mathsf{T}^{22}\end{bmatrix}\begin{bmatrix}\mathsf{p}\\ \mathsf{q}\end{bmatrix}, \tag{37}$$

or

$$\begin{aligned}p_{ml}&=\sum_{l'=1}^{N}\sum_{m'=-l'}^{l'}(T^{11}_{mlm'l'}a_{m'l'}+T^{12}_{mlm'l'}b_{m'l'}),\\ q_{ml}&=\sum_{l'=1}^{N}\sum_{m'=-l'}^{l'}(T^{21}_{mlm'l'}a_{m'l'}+T^{22}_{mlm'l'}b_{m'l'}),\end{aligned} \tag{38}$$

Note that in Eq. (36) the terms related to $\text{Re}(q_{ml}b^*_{ml}+p_{ml}a^*_{ml})$ and $|q_{ml}|^2+|p_{ml}|^2$ correspond to the extinction and scattering torques in Eq. (2), respectively. Each element of the T matrix has the general symmetric property [54],

$$T^{ij}_{-ml,-m'l'}=(-1)^{m+m'}T^{ji}_{m'l',ml}. \tag{39}$$

Substituting Eq. (38) into (36) yields,

$$\begin{aligned}T_z=&-\tfrac{\varepsilon}{2k^3}\sum_{l=1}^{N}\sum_{m=-l}^{l}\sum_{l'=1}^{N}\sum_{m'=-'l}^{l'}m\text{Re}(T^{21}_{mlm'l'}a_{m'l'}b^*_{ml}+T^{22}_{mlm'l'}b_{m'l'}b^*_{ml}+T^{11}_{mlm'l'}a_{m'l'}a^*_{ml}+T^{12}_{mlm'l'}b_{m'l'}a^*_{ml})\\ &-\tfrac{\varepsilon}{2k^3}\sum_{l=1}^{N}\sum_{m=-l}^{l}\sum_{l'=1}^{N}\sum_{m'=-'l}^{l'}(S^{21}_{mlm'l'}a_{m'l'}b^*_{ml}+S^{22}_{mlm'l'}b_{m'l'}b^*_{ml}+S^{11}_{mlm'l'}a_{m'l'}a^*_{ml}+S^{12}_{mlm'l'}b_{m'l'}a^*_{ml}),\end{aligned} \tag{40}$$

where $S^{ij}_{mlm'l'}$ represents the element of the matrix constructed by

$$\mathsf{S}=\begin{bmatrix}\mathsf{S}^{11} & \mathsf{S}^{12}\\ \mathsf{S}^{21} & \mathsf{S}^{22}\end{bmatrix}=\mathsf{T}^{\dagger}\mathsf{M}\mathsf{T},\ M_{mlm'l'}=m\delta_{mm'}\delta_{ll'}, \tag{41}$$

We notice that in Eq. (40) only $T^{ij}_{mlm'l'}$ and $S^{ij}_{mlm'l'}$ depend on the particle's orientation α. Then performing the angular integral or average over α for this equation, we have the nonconservative torque given by:

$$\begin{aligned} T_z^{\rm ncons} = &-\tfrac{\varepsilon}{2k^3}\sum_{l=1}^{N}\sum_{m=-l}^{l}\sum_{l'=1}^{N}\sum_{m'=-'l}^{l'} m\mathrm{Re}(\langle T^{21}_{mlm'l'}\rangle a_{m'l'}b^*_{ml} + \langle T^{22}_{mlm'l'}\rangle b_{m'l'}b^*_{ml} + \langle T^{11}_{mlm'l'}\rangle a_{m'l'}a^*_{ml} + \langle T^{12}_{mlm'l'}\rangle b_{m'l'}a^*_{ml}) \\ &-\tfrac{\varepsilon}{2k^3}\sum_{l=1}^{N}\sum_{m=-l}^{l}\sum_{l'=1}^{N}\sum_{m'=-'l}^{l'} (\langle S^{21}_{mlm'l'}\rangle a_{m'l'}b^*_{ml} + \langle S^{22}_{mlm'l'}\rangle b_{m'l'}b^*_{ml} + \langle S^{11}_{mlm'l'}\rangle a_{m'l'}a^*_{ml} + \langle S^{12}_{mlm'l'}\rangle b_{m'l'}a^*_{ml}), \end{aligned} \tag{42}$$

where $\langle\cdot\rangle$ denotes the angular average operator. Utilizing the Wigner D functions [54] and the identity, $\int_0^{2\pi} e^{-i(m-m')\alpha} = \delta_{mm'}$, one may obtain the following characteristics of the averaged matrix elements:

$$\begin{aligned} \langle T^{ij}_{mlm'l'}\rangle = \delta_{mm'}\langle T^{ij}_{mlm'l'}\rangle,\ \langle T^{ij}_{-ml,-m'l'}\rangle = (-1)^{i+j}\langle T^{ij}_{mlm'l'}\rangle, \\ \langle S^{ij}_{mlm'l'}\rangle = \delta_{mm'}\langle S^{ij}_{mlm'l'}\rangle,\ \langle S^{ij}_{-ml,-m'l'}\rangle = (-1)^{i+j+1}\langle S^{ij}_{mlm'l'}\rangle. \end{aligned} \tag{43}$$

With Eqs. (43), we can reduce the summations in Eq. (42) and recast it into a more symmetric form:

$$T_z^{\rm ncons} = -\frac{\varepsilon}{2k^3}\sum_{l=1}^{N}\sum_{m=0}^{l}\sum_{l'=1}^{N}(1-\frac{\delta_{m0}}{2})\mathrm{Re}\begin{bmatrix} (m\langle T^{21}_{mlml'}\rangle + \langle S^{21}_{mlml'}\rangle)(a_{ml'}b^*_{ml} + a_{-ml'}b^*_{-ml}) \\ +(m\langle T^{12}_{mlml'}\rangle + \langle S^{12}_{mlml'}\rangle)(b_{ml'}a^*_{ml} + b_{-ml'}a^*_{-ml}) \\ +(m\langle T^{22}_{mlml'}\rangle + \langle S^{22}_{mlml'}\rangle)(b_{ml'}b^*_{ml} - b_{-ml'}b^*_{-ml}) \\ +(m\langle T^{11}_{mlml'}\rangle + \langle S^{11}_{mlml'}\rangle)(a_{ml'}a^*_{ml} - a_{-ml'}a^*_{-ml}) \end{bmatrix} \tag{44}$$

Then the key step is to express a_{ml} and b_{ml} in terms of the incident fields. This can be done by taking advantage of the orthogonality among the VSHFs (cf. page 377 in ref. [54]):

$$a_{ml} = 4\pi(-1)^m i^l G_{ml}\mathbf{E}\cdot\mathbf{C}_{-ml}(\theta,\phi),\ b_{ml} = 4\pi(-1)^m i^{l-1} G_{ml}\mathbf{E}\cdot\mathbf{B}_{-ml}(\theta,\phi), \tag{45}$$

where

$$G_{ml} = \sqrt{\frac{(2l+1)(l+m)!}{4\pi(l+1)(l-m)!}}, \tag{46}$$

and the VSHFs are given by

$$\begin{aligned} \mathbf{B}_{lm}(\theta,\phi) = \left[\mathbf{e}_\theta\frac{d}{d\theta}P_l^m(\cos\theta) + \mathbf{e}_\phi\frac{im}{\sin\theta}P_l^m(\cos\theta)\right]e^{im\phi}, \\ \mathbf{C}_{lm}(\theta,\phi) = \left[\mathbf{e}_\theta\frac{im}{\sin\theta}P_l^m(\cos\theta) - \mathbf{e}_\phi\frac{d}{d\theta}P_l^m(\cos\theta)\right]e^{im\phi}. \end{aligned} \tag{47}$$

Plugging Eq. (5) in the manuscript into Eq.(45) yields the formulas:

$$\begin{aligned} a_{ml} = 2\pi E_0(-1)^m i^l G_{ml}\left[i(e^{iKx} - e^{-im\pi}e^{-iKx})\pi_{-m,l} + \cos\theta(e^{-im\pi/2}e^{iKy} + e^{im\pi/2}e^{-iKy})\tau_{-m,l}\right]e^{ik_z z}, \\ b_{ml} = 2\pi E_0(-1)^m i^{l-1} G_{ml}\left[(e^{iKx} - e^{-im\pi}e^{-iKx})\tau_{-m,l} - i\cos\theta(e^{-im\pi/2}e^{iKy} + e^{im\pi/2}e^{-iKy})\pi_{-m,l}\right]e^{ik_z z}, \end{aligned} \tag{48}$$

with

$$\tau_{ml} = \frac{d}{d\theta}P_l^m(\cos\theta),\ \pi_{ml} = \frac{m}{\sin\theta}P_l^m(\cos\theta). \tag{49}$$

Ultimately, with Eqs. (48), the identity for the associated Legendre polynomials $P_l^{-m}(\cos\theta) = (-1)^m\frac{(l-m)!}{(l+m)!}P_l^m(\cos\theta)$, and Eq. (7) in the main text, we are led to the following results for the traveling wave (i.e., real k_z):

$$\begin{bmatrix} (a_{ml'}b^*_{ml} + a_{-ml'}b^*_{-ml}) \\ (b_{ml'}a^*_{ml} + b_{-ml'}a^*_{-ml}) \\ (b_{ml'}b^*_{ml} - b_{-ml'}b^*_{-ml}) \\ (a_{ml'}a^*_{ml} - a_{-ml'}a^*_{-ml}) \end{bmatrix} \begin{cases} \propto\ \sin(Kx)\cos(Ky) \propto (\nabla\times\mathbf{p})_z & \text{for even } m \\ 0 & \text{for odd } m \end{cases}. \tag{50}$$

It follows from Eqs. (44) and (50) that $T_z^{\rm ncons}$ is proportional to the momentum curl $(\nabla\times\mathbf{p})_z$ for higher multipoles $(N>1)$. However, $T_z^{\rm ncons}$ should be zero on the dipoles $(N=1)$, for which $l=l'=1$ and $m=0,1$ in Eq. (44). This is because the matrix elements-related terms in Eq. (44) are zero at $l=l'=1$ and $m=0$; and for $m=1$ (odd), the expansion coefficients-related terms will vanish according to Eq. (50).

On the other hand, one may also work out the expansion coefficients for the evanescent wave (i.e., imaginary $k_z=iq$):

$$\begin{bmatrix} (a_{ml'}b^*_{ml}+a_{-ml'}b^*_{-ml}) \\ (b_{ml'}a^*_{ml}+b_{-ml'}a^*_{-ml}) \\ (b_{ml'}b^*_{ml}-b_{-ml'}b^*_{-ml}) \\ (a_{ml'}a^*_{ml}-a_{-ml'}a^*_{-ml}) \end{bmatrix} \begin{cases} \propto\ \sin(Kx)\cos(Ky)e^{-2qz}\propto(\nabla\mathcal{H})_z & \text{for even}\, m \\ 0 & \text{for odd}\, m \end{cases}, \tag{51}$$

where we have used Eq. (8) in the main text. Therefore, the nonconservative torque $T_z^{\rm ncons}$ on the higher multipoles is induced by the reactive helicity gradient $(\nabla\mathcal{H})_z$ for the evanescent wave. Likewise, this gradient torque should also be zero for the dipoles.

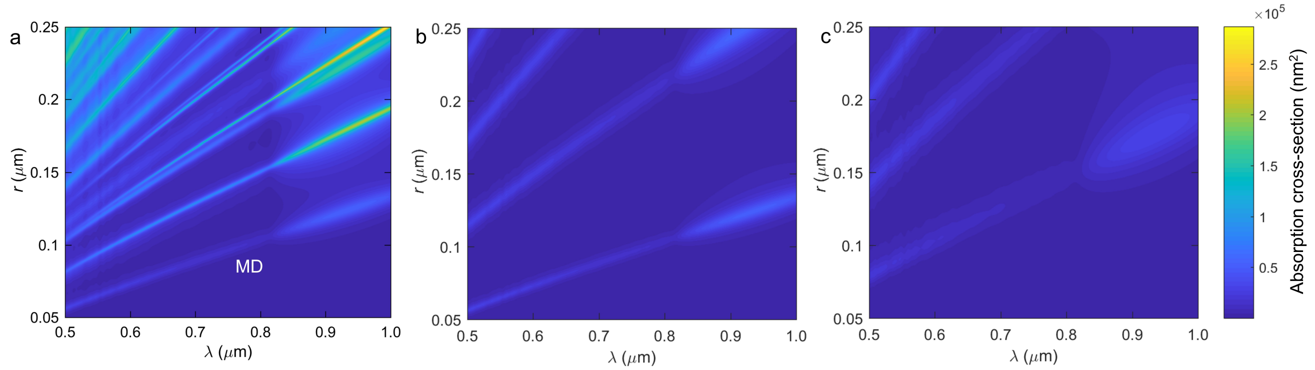

FIG. S1. Total absorption spectrum of Si particles (**a**) and the contributions from the magnetic (**b**) and electric (**c**) dipoles. One may notice that there is a significant resonance across the small radius region in **a**, which is mainly attributed to the magnetic dipole (**b**). However, it is evident that the NOT is absent in this area (cf. Figs. 2e,f), for the high-order nature of the curl and gradient torques.

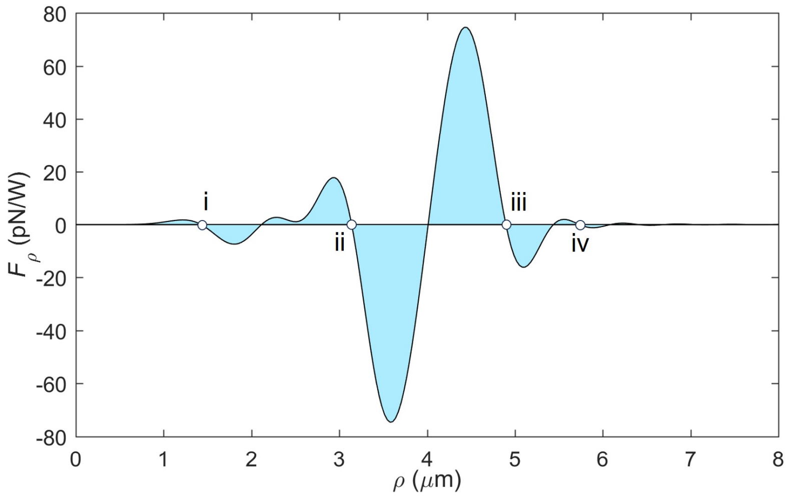

FIG. S2. Calculated radial force F_ρ for the Au particle used in Fig. 4b. Radial trapping occurs when $F_\rho = 0$ and $\partial F_\rho/\partial\rho < 0$, so four trapping positions (i-iv) can be identified. In particular, position iii ($\rho = 4.8$ μm) is located within the NOT region shown in Fig. 4b.

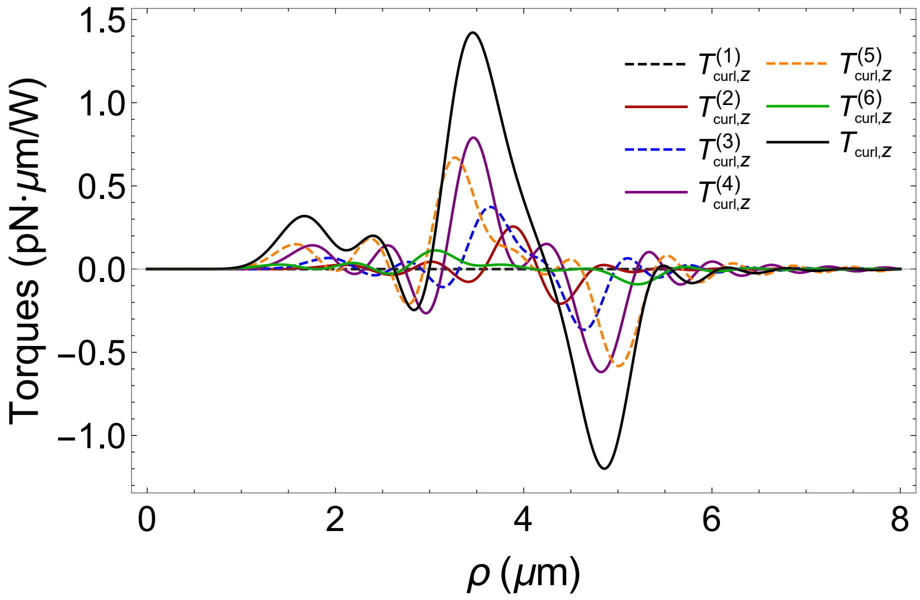

FIG. S3. Multipolar decomposition of the curl torque in Fig. 4b. The results are negligible for multipoles of orders higher than 6 (not shown). It is evident that the negative torque involves multipoles from the quadrupole to 2^6-pole. In particular, the torque at the trapping position ($\rho = 4.8$) μm is mainly contributed from the 2^4- and 2^5-poles.

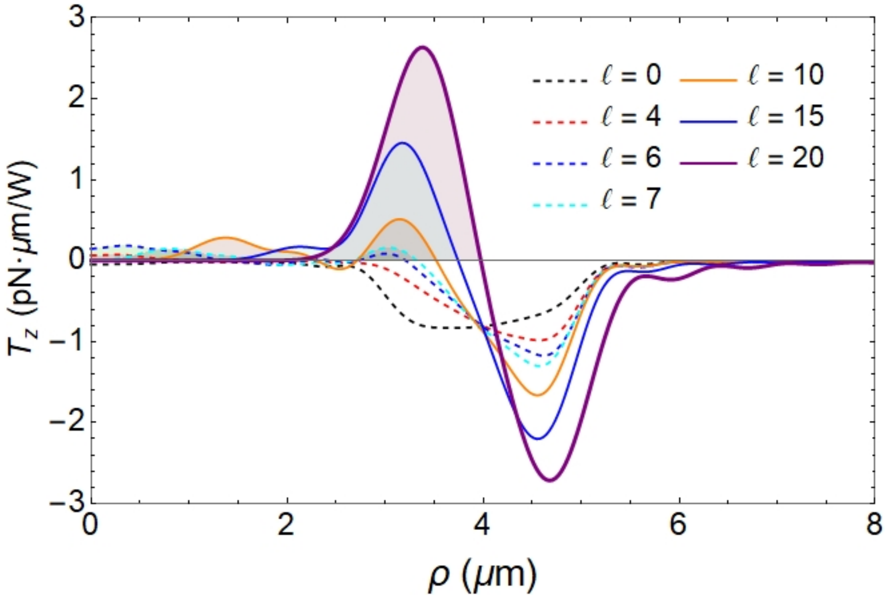

FIG. S4. Longitudinal optical torque calculated for right-handed circular polarization, which will generate negative spin. In this case, the NOT corresponds to positive torque values by definition.
